# A Unified Model of the GABA_A_ Receptor Comprising Agonist and Benzodiazepine Binding Sites

**DOI:** 10.1371/journal.pone.0052323

**Published:** 2013-01-07

**Authors:** Rikke Bergmann, Kristine Kongsbak, Pernille Louise Sørensen, Tommy Sander, Thomas Balle

**Affiliations:** 1 Department of Drug Design and Pharmacology, Faculty of Health and Medical Sciences, University of Copenhagen, Copenhagen, Denmark; 2 National Food Institute, Technical University of Denmark, Søborg, Denmark; 3 Novo Nordisk A/S, Bagsværd, Denmark; 4 Faculty of Pharmacy, The University of Sydney, Sydney, New South Wales, Australia; Russian Academy of Sciences, Institute for Biological Instrumentation, Russian Federation

## Abstract

We present a full-length α_1_β_2_γ_2_ GABA receptor model optimized for agonists and benzodiazepine (BZD) allosteric modulators. We propose binding hypotheses for the agonists GABA, muscimol and THIP and for the allosteric modulator diazepam (DZP). The receptor model is primarily based on the glutamate-gated chloride channel (GluCl) from *C. elegans* and includes additional structural information from the prokaryotic ligand-gated ion channel ELIC in a few regions. Available mutational data of the binding sites are well explained by the model and the proposed ligand binding poses. We suggest a GABA binding mode similar to the binding mode of glutamate in the GluCl X-ray structure. Key interactions are predicted with residues α_1_R66, β_2_T202, α_1_T129, β_2_E155, β_2_Y205 and the backbone of β_2_S156. Muscimol is predicted to bind similarly, however, with minor differences rationalized with quantum mechanical energy calculations. Muscimol key interactions are predicted to be α_1_R66, β_2_T202, α_1_T129, β_2_E155, β_2_Y205 and β_2_F200. Furthermore, we argue that a water molecule could mediate further interactions between muscimol and the backbone of β_2_S156 and β_2_Y157. DZP is predicted to bind with interactions comparable to those of the agonists in the orthosteric site. The carbonyl group of DZP is predicted to interact with two threonines α_1_T206 and γ_2_T142, similar to the acidic moiety of GABA. The chlorine atom of DZP is placed near the important α_1_H101 and the N-methyl group near α_1_Y159, α_1_T206, and α_1_Y209. We present a binding mode of DZP in which the pending phenyl moiety of DZP is buried in the binding pocket and thus shielded from solvent exposure. Our full length GABA_A_ receptor is made available as Model S1.

## Introduction

**Figure 1:**
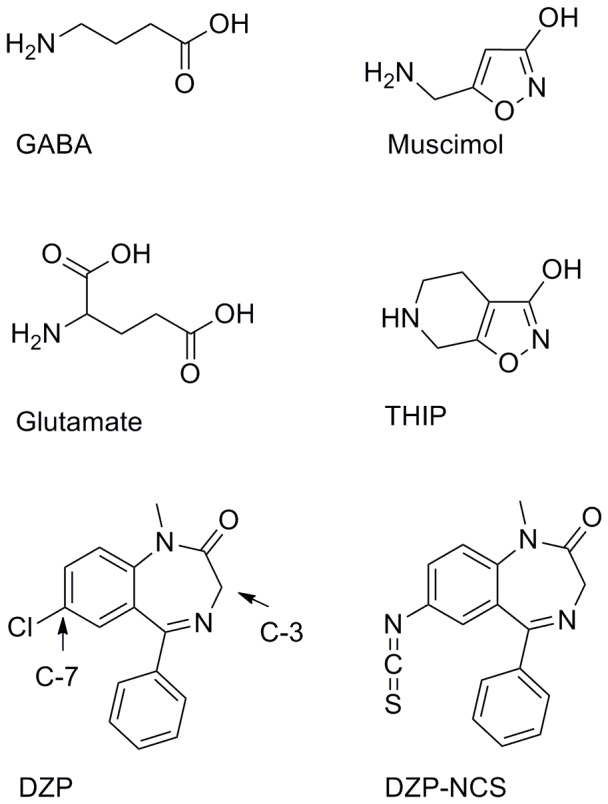
Some classical GABA_A_ receptor ligands. GABA is the endogenous GAB_A_R agonist, muscimol a classical high-affinity agonist and THIP a muscimol analogue. Although not a GAB_A_R ligand, glutamate is included to illustrate the resemblance to GABA. Diazepam (DZP) belongs to the benzodiazepine class of compounds, which are allosteric GABA_A_ modulators. The DZP-NCS analogue attaches covalently to GABA_A_Rs and is included for validation purposes.

**Figure 2:**
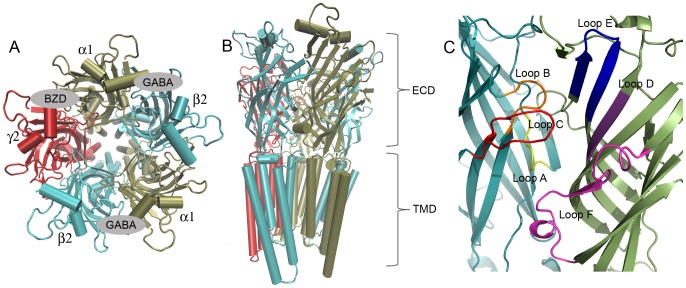
Illustration of the GABA_A_R structural composition. **A**) Top view showing the pentameric assembly of α_1_, β_2_ and γ_2_ subunits and the location of binding sites for GABA and BZDs; **B**) Side view illustrating the extracellular domain (ECD) where agonists and benzodiazepines bind and the transmembrane domain (TMD); **C**) Zooming in on a GABA binding site at the subunit interface between β_2_ and α_1_ subunits, loop regions A–F mentioned in the text are shown (A: yellow, B: orange, C: red, D: purple, E: blue and F: pink).

γ-aminobutyric acid (GABA) is the major inhibitory neurotransmitter in the central nervous system (CNS) as opposed to glutamic acid, which is the primary excitatory CNS-neurotransmitter (Figure 1). Structurally, the two compounds are similar, and in fact GABA is formed *in vivo* by decarboxylation of glutamate. GABA_A_ receptors (GABA_A_Rs) are involved in a number of important functions such as cognition, learning, and memory and in disorders such as epilepsy, anxiety, schizophrenia, sleep disorders, and depression [1]. The GABA_A_Rs belong to the Cys-Loop receptor family that also includes nicotinic acetylcholine receptors (nAChRs), serotonine type 3 receptors (5-HT_3_Rs) and glycine receptors (GlyRs). All Cys-Loop receptors are homomeric or heteromeric assemblies of five subunits forming a central ion-conducting pore (Figure 2). The GABA_A_Rs and GlyRs conduct anions whereas nAChRs and 5-HT_3_Rs are cation selective. Each subunit is made up of an extracellular domain (ECD) consisting of mainly β-sheets, and a trans-membrane domain (TMD) consisting of four membrane spanning α-helices. GABA_A_R subunits include α_1–6_, β_1–3_, γ_1–3_, δ, ε, π, θ, ρ_1–3_ and the most abundant GABA_A_R subunit combination in the human CNS is the α_1_β_2_γ_2_ subtype where the endogenous neurotransmitter GABA binds in each of the interfaces between β_2_ and α_1_ subunits (Figure 2). A modulatory site for benzodiazepine (BZD) like compounds is found in a homologous position between α_1_ and γ_2_ subunits.

Despite decades of research and a wealth of experimental and theoretical studies, the exact binding mode of key agonists including GABA is still unknown. The same is the case for the BZDs. Key agonists for the GABA binding site include the high affinity agonist muscimol [2], [3] and the partial agonist THIP [4], [5], which is a structurally restrained muscimol analog (Figure 1). THIP was long in clinical trials for treatment of insomnia, but was discontinued. Still, the GABA_A_R agonist binding site is regarded a promising drug target and represents an intriguing alternative to the BZD binding site, which has long been the target for allosteric modulators including BZDs such as diazepam (Figure 1). BZDs are still one of the most prescribed classes of drugs for the treatment of insomnia, anxiety, and convulsions [6], [7].

So far, drug discovery efforts have relied mainly on indirect structural insight from focused [8]–[12] or unified pharmacophore models recapitulating the structure-activity relationships (SAR) of compounds synthesized during more than fifty years of active medicinal chemistry research in the field [13], [14]. Homology models, on the other hand, have had little practical impact on the design process despite a number of models reported in the literature [15]–[25]. The models were mainly built using the homologous acetylcholine binding proteins (AChBPs) as templates. The AChBPs have supplied insight into a number of structural features of Cys-Loop receptors. The position of loops A–F (Figure 2) known from mutational studies to participate in ligand binding were established with the first AChBP structure [26]. A high degree of flexibility has later on been observed for the C-loop, which is a hair-pin shaped loop that embraces the orthosteric binding sites and shields from the solvent [27]. It was observed that depending on the type of ligand in the binding site, the C-loop either exists in a closed (agonist) conformation or an open (antagonist) conformation allowing large inhibitors to enter the binding site. This C-loop movement has also been speculated to be linked to the activation mechanism of Cys-Loop receptors [28].

Although, the AChBPs have proven valuable templates for modeling of nAChRs [26], [29]–[32] they suffer from a lack of conservation of binding site residues with respect to GABA_A_Rs, which makes them unsuitable as stand-alone templates for GABA_A_R homology modeling. To compensate for the lack of conservation of binding site residues, we have recently reported a novel strategy for GABA_A_R modeling utilizing experimental restraints and multiple templates including AChBPs from different species [27], [33], a mouse α_1_ nAChR subunit [34], and the bacterial orthologs from Gloeobacter violaceus (GLIC) [35] and Erwinia chrysanthemii (ELIC) [36] in the alignment generation and model building steps [37]. In particular, inclusion of the ELIC structure adds important conserved binding site residues to the pool of template structures. Using this strategy, a reliable model of the GABA_A_R ECD with focus on the orthosteric ligand binding interface of the α_1_β_2_γ_2_ GABA_A_R in its non-activated (antagonized) state was obtained. The model was consistent with experimental data and capable of rationalizing the structure activity relationships (SAR) of a series of GABA_A_R orthosteric antagonists [37].

With the recent release of atomic resolution structures of a eukaryotic glutamate gated chloride channel (GluCl) [38] from the nematode *C. elegans*, the molecular basis for modeling of pentameric ligand gated anion channels has improved considerably. The GluCl structure has an unprecedented high sequence identity compared to the GABA_A_R; 30%, 36%, and 31% relating to α1, β2 and γ2 subunits, respectively, and even higher identities with respect to the ligand binding cores (∼48% in an 8 Å radius from glutamate in GluCl). The GluCl structure was crystallized in presence of its agonist glutamate and was captured in its presumed open state.

In this report we demonstrate the use of the GluCl structure as template for construction of a GABA_A_ receptor homology model comprising both the ECD and TMD portions of the receptor. We show that when *combined* with the structure of the bacterial ELIC channel, a reliable GABA_A_R model based entirely on full length receptor X-ray structures can be obtained. The model is built in the open state with GABA in the two orthosteric binding sites between β_2_ and α_1_ subunits. The BZD binding site between the α_1_ and γ_2_ subunit is adapted to the positive allosteric modulator diazepam (DZP). The model is capable of explaining SARs, mutational data, and data from studies of covalent linking of a DZP-derivative to cysteine mutants of the receptor. Therefore, the validated model might also serve as a tool for structure guided design of new agonists and allosteric modulators and may form the link to interpretation of previously reported pharmacophore models [11], [14], [39] in a structural context.

## Methods

### Homology modeling

#### Templates, sequences and sequence alignment

**Figure 3:**
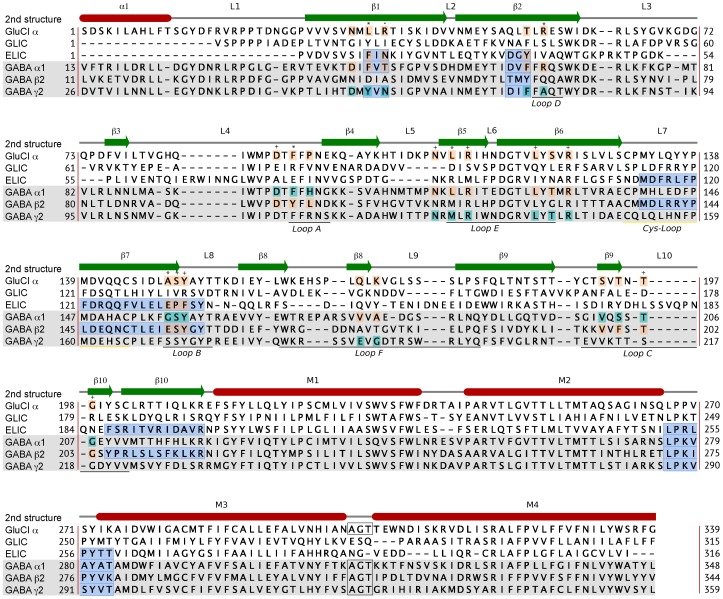
Alignment of protein sequences from GluCl, GLIC, ELIC and the human α_1_, β_2_, and γ_2_ GABA_A_R subunits. The GluCl sequence was used as template for homology modeling throughout the GABA_A_R subunits, and ELIC was included as a template in the regions marked with blue boxes. The secondary structure deduced from the X-ray structure of GluCl is shown above the alignment (red shapes denote α-helices, and green arrows denote β-strands), whereas historically assigned loop regions are indicated below the alignment. As in the GluCl structure the M3–M4 intracellular loop of GABA_A_R sequences was replaced by an AGT tri-peptide linker. Residues comprising the binding sites (within 8 Å of Glu in the GluCl structure and pointing towards the binding site) are colored pink (Glu and GABA binding site) and cyan (BZD binding site). Binding site residues conserved with respect to the templates are indicated as follows: **+** conserved in both GABA and BZD binding sites; * conserved in the GABA binding sites; **.** conserved in the BZD binding site. For details on calculation of binding site sequence identities see Figure S1.

The X-ray structure of GluCl co-crystallised with glutamate (PDB code 3RIF) [38] was used as primary template for homology modeling of the most abundant subtype of the GABA_A_R, α_1_β_2_γ_2_. In a few important regions with low sequence identity to GluCl the bacterial homologue ELIC (PDB code 2VL0) [40], which has a 20% sequence identity to GluCl, was included as template as well. The extent to which each template structure was used is specified in Figure 3, in which the definitions for the general secondary structural elements of Cys-Loop receptors referred to throughout this paper are also indicated. The rationale for including ELIC as template in the areas highlighted in Figure 3 were the following: 1) In the β1 and β2 sheets the ELIC structure contains aromatic residues in positions 19 and 38 resembling those in the GABA_A_R; 2) In the β6–β7-loop (Cys-Loop) and in the β7 and β10 strands ELIC was included as template for the β_2_ subunit to capture information about the conformations of and interactions between GABA_A_R β_2_E155 and β_2_R207; 3) In the M2–M3-loop ELIC was included as template due to the presence of Pro residues in homologous positions.

The sequence alignment was obtained following the procedure reported by *Sander et al.* [37] First, a structural alignment of the two template structures was generated using Pymol 1.3 [41]. Subsequently, all human GlyR α-subunits and all human GABA_A_R subunits were aligned to the GluCl sequence as profile alignments with iteration on the last alignment using ClustalX v. 2.0.12 [42]. The GlyR α-subunits represent the closest human homologs to GluCl and were included to aid identification of semi-conserved motifs. In three regions, namely, 1) in and after the N-terminal α-helix, 2) in loop F, and 3) in loop C, manual adjustments of the generated alignment were performed to ensure proper alignment of conserved motifs. 1) In the N-terminal α-helix the motif represented by the GABA_A_ α_1_ sequence ILDRLLDGYDNRLRP was misaligned by ClustalX due to the presence of insertions in the GlyR α-subunit sequences and the GABA_A_R ρ-subunit sequences. Therefore, this motif was reestablished as described by Sander *et al*. [37] 2) In loop F varying sequence lengths and low sequence identity resulted in a poor alignment and many gaps. We identified a hydrophobic-X-hydrophobic motif (corresponding to VVV in the GABA_A_R α_1_-subunit), forming a short β-strand of three residues in GluCl, ELIC, the bacterial ion channel GLIC (PDB ID: 3EAM) the mouse nAChR α_1_-subunit (PDB ID: 2QC1) and in AChBPs from Aplysia californica (PDB ID: 2BYQ) and Bulinus truncatus (PDB ID: 2BJ0) [27], [34], [35], [43]. The generated alignment was manually adjusted to re-establish this motif in the GABA_A_R sequences. 3) In loop C, the automatically generated alignment from ClustalX had gaps in the GABA_A_R sequences in the β-sheet regions. These were manually moved to the tip of the loop as it is generally accepted that the length of loop C varies between families and subtypes of Cys-Loop receptors. 4) Finally, we truncated the M3–M4 intracellular loop and inserted an AGT tripeptide according to the GluCl structure. The manually adjusted alignment is reported in Figure 3.

Prior to model building the GluCl X-ray structure was prepared as follows. The FAB fragments (chains F–O) as well as all heteroatoms were removed except glutamates in the orthosteric binding sites between chains A, B and C, D. Then the α-carboxylic acid moiety was deleted from the glutamate ligands, resulting in a GluCl template structure with GABA in the two orthosteric binding sites between chains A, B and C, D.

#### Model building, evaluation and selection

The program MODELLER 9v7 [44] was used for homology modeling using the “automodel class”, which includes no other restraints than spatial restraints gathered from the sequence alignment. 100 models were generated, and the refinement level “refine.slow” was applied. GABA molecules were modeled into the two β_2_-α_1_ subunit interfaces in the GABA_A_ receptor model as rigid bodies.

The final model selection was performed according to the consensus scoring approach described by Sander *et al.* [37] using the ProSA z-score [45], the energy according to the OPLS 2001 force field [46], [47] as implemented in Maestro [48], and the MODELLER built in scoring functions, *molpdf* and DOPE score [44]. The consensus 10 best scoring models were assessed visually for physico-chemical requirements such as packing of hydrophobic residues in hydrophobic environments and solvent exposure of charged residues. Also, the interactions between the modeled GABA molecule and the receptor model were assessed as part of the selection criteria.

### Model refinement

The selected model was subjected to the protein preparation wizard in Maestro [48], which adds hydrogen atoms, assigns bond orders, creates disulphide bonds and samples hydrogen bond networks. Furthermore, the protein preparation wizard assesses the protonation state of His, Lys, Arg, Glu, and Asp. As is seen in the GluCl structure E293 interacts with R245 and D316, indicating that this residue exists in its protonated form. Therefore, the corresponding five Glu residues (two α_1_E302, two β_2_E298, and one γ_2_E313) in the homology model, which also coordinates to Arg and Asp were protonated. The protein preparation wizard and the PROPKA web server [49]–[51] further supported this assessment. All other residues were kept at their standard protonation states (neutral His, protonated Lys and Arg and deprotonated Glu and Asp). Finally, an energy minimization with a flat-bottomed Cartesian constraint and a convergence threshold set to an RMSD of 0.3 Å was performed.

The model was further refined as follows: 1) The rotameric state of α_1_R66 (in chain D) was optimized for optimal bidentate interactions with GABA using the side chain refinement tool in Prime [52]; 2) Hydrogen bond networks between the GABA molecules and the receptor model were manually optimized by selecting appropriate rotamers of α_1_T129 similar to the homologous S121 in the GluCl structure; 3) Loop A (residues 99–102) in the BZD site carrying α_1_H101 was sampled using the loop sampling protocol in Prime [52] in order to obtain an orientation of α_1_Asn102 in agreement with the template structure; 4) A rotamer of β_2_K196 able to make a salt bridge with β_2_E153 was selected (Table 1).

**Table 1:** Overview of mutations affecting the function of the GABA_A_R or the binding of orthosteric ligands.

Residue	Proposed function/feature	Reference(s)
Structural features		
β_2_E153, β_2_K196	Intra-subunit salt bridges important for receptor function.	[112]
Ligand-binding features		
α_1_F64	Affects binding of bicuculline and gabazine and SCAM identified residue to be part of binding site.	[84]–[86], [113]
α_1_R66	Identified by SCAM to be part of binding site.	[84], [86]
α_1_L117	Identified by SCAM to be part of binding site. Also binding of gabazine is affected.	[89]
α_1_R119	Predicted by SCAM to line binding site. Mutation to Lys results in 180 fold reduced EC_50_ for GABA and inability of muscimol and gabazine to bind.	[89], [114]
α_1_T129	Predicted by SCAM to line binding site.	[85], [89]
α_1_R131	Predicted by SCAM to line binding site.	[89]
β_2_Y97	Identified by SCAM to be part of binding site. Artificial amino acid mutagenesis indicates participation in pi-cation interaction.	[91], [115]
β_2_E155	Cys mutation gives rise to spontaneously open channel. Predicted to be connected to gating and ligand binding.	[76]
β_2_Y157	Mutagenesis to Cys, Asn, Phe, and Ser indicate that this residue must be aromatic. Artificial amino acid mutagenesis does not indicate pi-cation interaction with this residue.	[76], [81], [91]
β_2_F200	Cys mutation significantly affects gabazine affinity as well as receptor activation.	[82]
β_2_S201	Cys mutation affects gabazine affinity as well as receptor activation.	[82]
β_2_T202	Ala and Cys mutations renders receptor essentially inactive. Ser mutation is accepted but decreases EC_50_ slightly.	[81], [82]
β_2_G203	Cys mutation severely affects affinity of gabazine and activation of the recptor.	[82]
β_2_Y205	Crucial residue that must be aromatic. Mutations to Ser and Asn renders the receptor inactive, and mutation to Cys affects both gabazine binding and receptor activation very severely.	[81], [82]
β_2_R207	Affects GABA binding and un-binding rates. Predicted to be part of binding site.	[78], [82]

All data in the table are in agreement with our GABA_A_R model.

### Ligand docking and binding site characterization

GABA, muscimol, and THIP were created in their ionized states in Maestro 9.2 [53] followed by conformational searches with MacroModel 9.9 (default settings) [54]. The global energy minimum conformations were identified and used as input conformations for docking. The agonists were docked into the orthosteric binding site between chains A and B using the Glide Induced Fit Docking (IFD) protocol [55], [56] and the Extra Precision (XP) scoring function [57]. By default the IFD procedure allows amino acid side chains to adapt to the docked ligand in a 5 Å sphere. Docking poses were selected based on compliance with mutational data (Table 1) and common interaction patterns in the binding site. Finally, selected ligand poses including residues in an 8 Å sphere were energy minimized to convergence using MacroModel 9.9.

The program GRID [58], [59] was used to characterize the non-bonded water interaction properties of the vacant binding pocket between chains A and B (GABA site) of the refined model using the water probe (OH2). A grid spacing of 0.33 Å was used and all other settings were kept at their default values.

Quantum mechanical (QM) calculations was performed using Jaguar 7.8 [60]. For muscimol, a relaxed coordinate scan was performed to determine conformational energies when varying the amino-methyl side chain dihedral angle in a step size of 10° between 0–180°. The Poisson-Bolzmann aqueous solvation model [61] and otherwise default settings were selected (B3LYP/6-31G**). Gas-phase energies were extracted from the results in order to consider only the steric energies.

The docking procedure described for the agonists was also attempted for DZP at the BZD site, but none of the obtained poses could be rationalized by experimental data. DZP was docked using its assumed bioactive conformation as input for docking [62]–[66]. QM partial charges were calculated using Jaguar 7.6 default settings. Subsequently, DZP was manually docked to the BZD binding site according to experimental evidence: Docked DZP should 1) have the Cl-substituent positioned in the vicinity of or pointing towards α_1_H101, α_1_N102 [67], α_1_G157, α_1_V202, and α_1_V211 [68], 2) have the C-3 atom positioned in the vicinity of or directed towards α_1_S205 and α_1_T206 [69], 3) have the *N*-methyl substituent directed towards an exit from the binding cavity [70], and 4) have the pending phenyl ring positioned in a lipophilic cavity [71], [72]. Following manual positioning of DZP, the α_1_-γ_2_ interface was allowed to adapt to DZP using the side chain prediction tool in Prime in which backbone and residue sampling within 4 Å of DZP was performed. As a final step, Prime performs a minimization of the complex, the docked ligand and the surrounding residues in question (backbone and side chains). The protein and the ligand were thus allowed to adapt to each other. The final model was further validated using 1) mutational studies from the literature (Table 2), 2) *in silico* covalent docking of a Cys-reactive DZP derivative, and 3) assessment of SAR data from the literature in a structural context.

**Table 2:** Overview of mutations affecting the function of the GABA_A_R or the ability of BZD binding site ligands to bind to the BZD binding site.

Residue	Proposed function/feature	Reference(s)
Ligand-binding features		
α_1_F99	May be involved in π-π stacking or might in some other way interact directly with ligands of the BZD type.	[67], [96]
α_1_H101	May be involved in π-π stacking or might in some other way interact directly with ligands of the BZD type.	[73], [97], [116]
α_1_Y159	Mutation to Ala, Cys or Ser severely affects binding affinity of BZDs. Residue lines the binding site.	[67], [68], [98]
α_1_G200	This residue lines a distal part of the binding site	[96], [104], [105], [116]
α_1_V202	This residue lines a distal part of the binding site	[96], [104]
α_1_T206	Mutation to Ala, Val, and Cys severely affects binding affinity of BZD-site ligands.	[68], [69], [99], [102], [105]
α_1_G207	Mutation to Cys severely affects binding affinity of flunitrazepam.	[68]
α_1_Y209	Aromatic functionality of the residue at this position is required for high-affinity binding of BZD binding site ligands.	[96], [98], [99]
γ_2_F77	Aromatic functionality of the residue at this position is required for high-affinity binding of BZD binding site ligands.	[100]–[102]
γ_2_M130	This residue lines the binding site	[96], [102]
Covalently modified mutants		
α_1_G157	7-NCS-derivatives of imidazo-BZDs react covalently with Cys-mutants of this residue suggesting the 7-position of imidazo-BZDs points towards this residue.	[68]
α_1_Y159	The NCS-derivative of imidazo-BZD does not react covalently with Cys-mutants of this residue. 7-substituent is not directed towards this residue.	[68]
α_1_V202	7-NCS-derivatives of imidazo-BZDs react covalently with Cys-mutants of this suggesting the 7-position of imidazo-BZDs points towards this residue.	[68]
α_1_S205	The 3-NCS-derivative of flunitrazepam reacts covalently with the Cys-mutant, forming a constitutively positively modulated receptor.	[69]
α_1_S206	The 3-NCS-derivative of flunitrazepam reacts covalently with the Cys-mutant, forming a constitutively positively modulated receptor.	[69]
α_1_V211	7-NCS-derivatives of imidazo-BZDs react covalently with Cys-mutants of this residue suggesting the 7-position of imidazo-BZDs points towards this residue.	[68]

All data in the table is in agreement with our GABA_A_R model.

As a validation of the DZP binding mode, the Cys-reactive BZD derivative DZP-NCS [67], [68], [73], [74] (Figure 1) was covalently docked to an α_1_H101C variant of the homology model using the “covalent docking” module in Prime. The covalent docking module works by eliminating two atoms in order to form a new bond between the reacting molecules/species. Since Prime cannot handle simultaneous reduction of a double bond and formation of a new bond, the isothiocyanato group of DZP-NCS was reduced to a methanethioamide group prior to submission of the job. The thiol-hydrogen was defined as the leaving receptor atom. The conformation of the attachment residue was sampled and all other residues were kept fixed.

Finally, a 48 ns molecular dynamics simulation was performed to assess the stability of the final GABA_A_R model. Details are supplied as Model S1.

## Results and Discussion

With the improved structural templates available from efforts in structural biology it is now possible to build α_1_β_2_γ_2_ GABA_A_R models based entirely on full length receptor templates. We have created a full-length α_1_β_2_γ_2_ GABA model mainly based on the glutamate bound GluCl X-ray structure and partly using the bacterial Cys-Loop homolog ELIC as an additional template. The model has been optimized in the GABA and BZD binding sites for the agonists GABA, muscimol and THIP and the modulator diazepam. The GluCl X-ray structure with glutamate bound was crystallized in an open state with a negatively charged ion in the lower part of the TMD [38]. The ELIC structure, on the other hand, was crystallized in a putatively closed state in absence of a bound ligand [36]. Since only a few residue positions in our model have been modeled based on the ELIC structure, the overall architecture of the α_1_β_2_γ_2_ GABA model is obtained from the GluCl structure. Therefore, we regard our model as being in the open state.

### Model assessment

The pentameric GABA_A_R α_1_β_2_γ_2_ ECD-TMD homology model comprised 1676 residues distributed with 335, 334, and 333 residues in α_1_, β_2_, and γ_2_ GABA_A_R subunits respectively. The selected model had good backbone geometry with 98.8% of the residues in favorable or additionally allowed regions in the Procheck v. 3.5.4 Ramachandran plot [75]. All residues in disallowed regions in the Ramachandran plot were situated in solvent exposed loop regions distant to the binding site. The ProSA z-scores for the selected homology model were within the accepted area for X-ray structures from the PDB. Furthermore, stability of the model was assessed by a 48 ns molecular dynamics calculation which showed essentially no drift after termination of the equilibration protocol (see Figure S2).

#### Validation by mutational data

Both the GABA and BZD binding sites have been heavily investigated by site-directed mutagenesis (Tables 1 and 2). Among these mutations, some have been used to suggest which residues line the binding sites, which residues interact directly with different ligands, and which are regarded as important structural features of the receptor. These experimental data, including those listed in Tables 1 and 2 that were not directly imposed during model refinement, are explainable by our homology model with the suggested poses of GABA and DZP as described below.

### Agonist binding model

#### GABA

**Figure 5:**
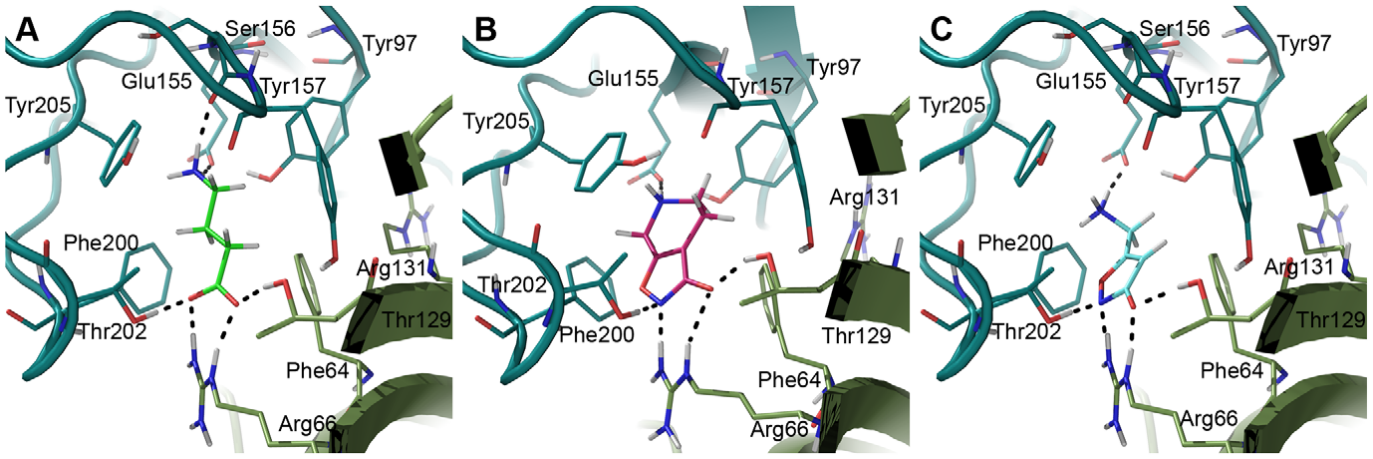
Agonist binding modes determined by induced fit docking. A) GABA (green), B) THIP (pink) and C) muscimol (cyan) are shown in the orthosteric binding site at the interface between the β_2_ subunit (teal) and the α_1_ subunit (smudge).

The X-ray structure of GluCl with glutamate bound presents a good indication of how GABA would bind to the GABA receptors. Our α_1_β_2_γ_2_ GABA model confirms that a similar GABA binding mode interacts well with the receptor and is in agreement with the experimental mutational data presented in Table 1. In this binding mode GABA forms salt bridges with α_1_R66 and β_2_E155 and hydrogen bonds with α_1_T129, β_2_T202, and the backbone of β_2_S156. Finally, there is a π-cation interaction with β_2_Y205. The GABA binding mode from our docking study is illustrated in Figure 5A. As described in the methods section GABA was modeled into the binding pockets of our receptor model as rigid bodies, which allowed space for GABA in the binding site. However, hydrogen bonding network was not optimized in the modeling process, hence, a few side chains needed adjustments as described in the methods section.

**Figure 4:**
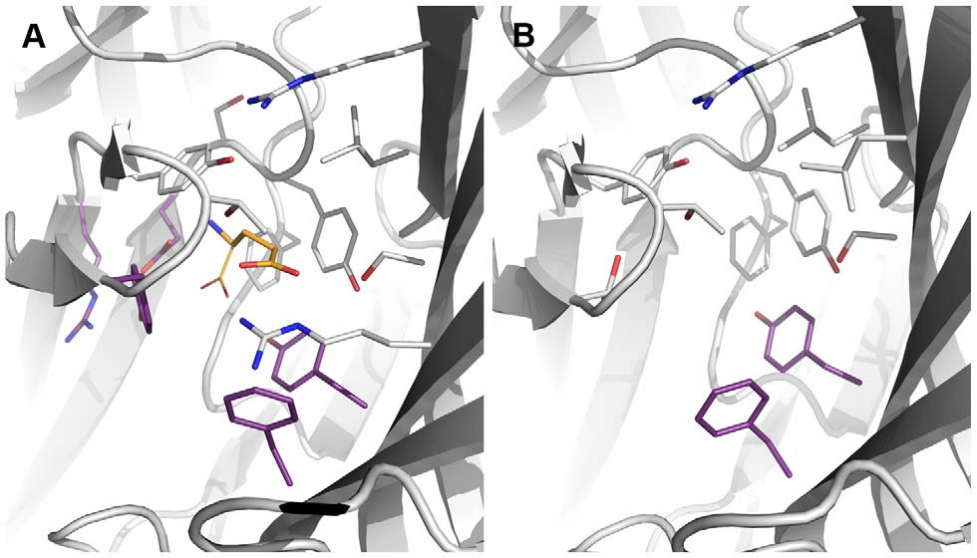
Conserved template residues. The figure shows residues that are conserved or homologous to GABA_A_R binding site residues from the GluCl X-ray structure (PBD ID 3RIF) as grey sticks and the bacterial Cys-Loop receptor homolog, ELIC (PDB ID 2VL0) as purple sticks. Glutamate as co-crystallized with GluCl is shown in yellow, where the structure corresponding to GABA is shown as sticks and the α-carboxylic acid removed prior to homology modeling is shown as lines. A) GABA binding site and B) BZD binding site.

The GABA_A_ orthosteric binding site apparently resembles the GluCl glutamate binding site to a large extent. However, there are a few crucial differences. Newell *et al.* [76] identified β_2_E155 to be vital for GABA_A_ ligand binding and channel gating. This residue is lacking in the GluCl receptor, but is present in ELIC (Figure 4A), a receptor, which is known to be activated by e.g. GABA [77], an important reason for including the ELIC structure as modeling template. The position of β_2_E155 in our receptor model is in perfect hydrogen bonding distance to the protonated amine of GABA, thereby forming a salt bridge deeply buried in the pocket and surrounded by a number of aromatic residues (so-called aromatic box). β_2_E155 is flanked by β_2_R207 intruding from outside the pocket. This residue has also been investigated experimentally and shown to affect GABA binding and un-binding [78], which makes sense if GABA binds as predicted here, since there is a clear electrostatic interaction between β_2_R207 and GABA through β_2_E155. Two other important residues that are non-conserved between GluCl and GABA_A_, but present in ELIC, are the β_2_F200 (C-loop) and α_1_F64 (D-loop). They serve as components of the aromatic box, taking part in shielding the positive charge in an enclosure of aromatic planes.

When GABA was re-docked into the binding site, a similar binding pose was identified (Figure 5A). This extended conformation of GABA has previously been determined by X-ray crystallography [79], [80] and by conformational search found as one of several low energy conformations. However, the GABA alkyl chain is quite flexible, and it is likely that it is not entirely fixed in the protein bound state. The distance between the two charges is approximately 5 Å in the identified pose. However, even if the GABA alkyl chain should be slightly bent, resulting in a shorter inter-charge distance, optimal interactions can be obtained in the binding pocket by GABA interacting with the backbone carbonyl of β_2_Y157 in place of β_2_S156. Indeed, previous pharmacophore models disagree on which should be the exact charge-charge distance in GABA_A_ agonists. However, it has generally been proposed to be in the range 4–5 Å [11] and in fact, our GABA_A_R model predicts that agonist with different inter-charge distances (4–5 Å) may bind equally well to the receptor. Apart from the salt bridge to β_2_E155, the positive charge of GABA is further surrounded by the aromatic ring of β_2_Y205 and makes a hydrogen bond to the backbone β_2_S156 (B-loop) similar to glutamate in the GluCl template structure. Mutational data show that β_2_Y205 is crucial for binding gabazine, a selective, competitive GABA_A_ antagonist, and for channel gating and that this residue must be aromatic for the receptor to be functional (Table 1) [81]–[83]. The carboxylic acid of GABA is fixed between β_2_ and α_1_ subunits by a bidentate interaction with α_1_R66 and hydrogen bonds with the two threonines β_2_T202 (C-loop) and α_1_T129. Again, these interactions make perfect sense in the light of published experimental data (Table 1). Mutations of α_1_T202 have a crucial impact on GABA activation of the GABA_A_ receptor. Mutation to Ala or Cys renders the receptor virtually inactive, while some function is retained with a α_1_T202S mutation [81], [82]. α_1_R66C mutations have shown a 300–500 fold decrease in EC_50_ and the residue was shown by the substituted cysteine accessibility method (SCAM) to be part of the binding pocket [84], [85]. This residue has long been suspected to interact with the carboxylic acid of GABA [84], [86]–[88]. However, α_1_R119 (in GABAρ1), α_1_R131 and β_2_R207 have also been hypothesized to perform this interaction [78], [87], [89]. Based on the GluCl X-ray structure and available mutational data, the evidence for α_1_R66 to be the arginine interacting with the GABA carboxylic acid is quite strong. β_2_R207 interacts with GABA through β_2_E155 as discussed above. In our model α_1_R119 (conserved in GluCl) has an important structural role, as it links the α-subunit to the C-loop of the β-subunit (hydrogen bond to the backbone of β_2_T202) thereby forming a “roof” on top of the agonist binding site as well as an enforced closed state of the C-loop. A closed C-loop has long been regarded necessary for obtaining the active state of Cys-Loop receptors [27], [43], [90]. α_1_R131 is found lining the back wall of the binding site behind α_1_F64, where it interacts with α_1_D62 and the backbone of β_2_D101. Nevertheless, the flexible α_1_R131 could also reach β_2_Y97 to form a π-cation interaction. This residue was identified by *Padgett et al.* [91] to participate in a π-cation interaction in the GABA_A_ receptors and they suggested that the cation should originate from the GABA molecule. However, based on our model a π-cation interaction between GABA and β_2_Y97 is unlikely. Indeed, it has generally caused difficulty to generate a GABA_A_ receptor model with the aromatic ring of β_2_Y97 facing the binding pocket, which has not changed with the improved template GluCl [37], [91]. The authors suggested α_1_R131 as an alternative cation source in the binding site, and this would fit our model. The mutational consequences of β_2_Y97C and α_1_R131C could instead originate from perturbation of the activation mechanism.

#### THIP and muscimol

The docked poses of the other hallmark agonists THIP and muscimol are illustrated in Figure 5B–C. Poses with similar interactions as described above for GABA were identified, however, with some differences as discussed below.

The rigid THIP is able to make the same interactions as GABA. The 3-hydroxy-isoxazole moiety, a bio-isostere of the carboxylic acid in GABA, interacts with α_1_R66, β_2_T202 and α_1_T129 similar to GABA. Furthermore, the protonated amine forms a salt bridge to β_2_E155 and a pi-cation interaction with the important β_2_Y205.

**Figure 6:**
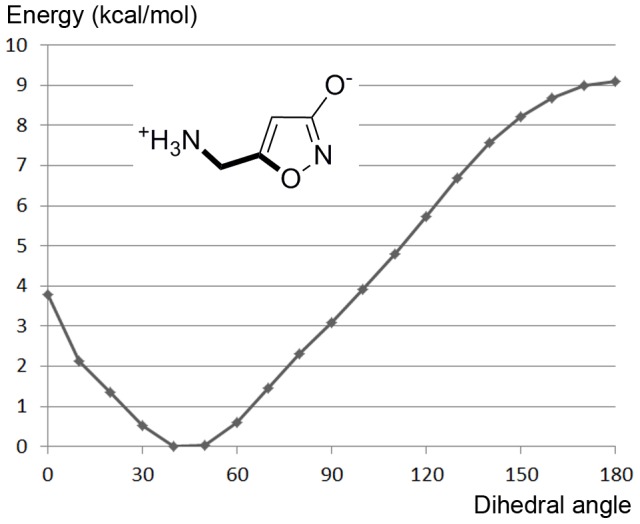
Conformational energy profile for dihedral drive of the amino-methyl side chain of muscimol. B3LYP/6-31G** energies.

**Figure 7:**
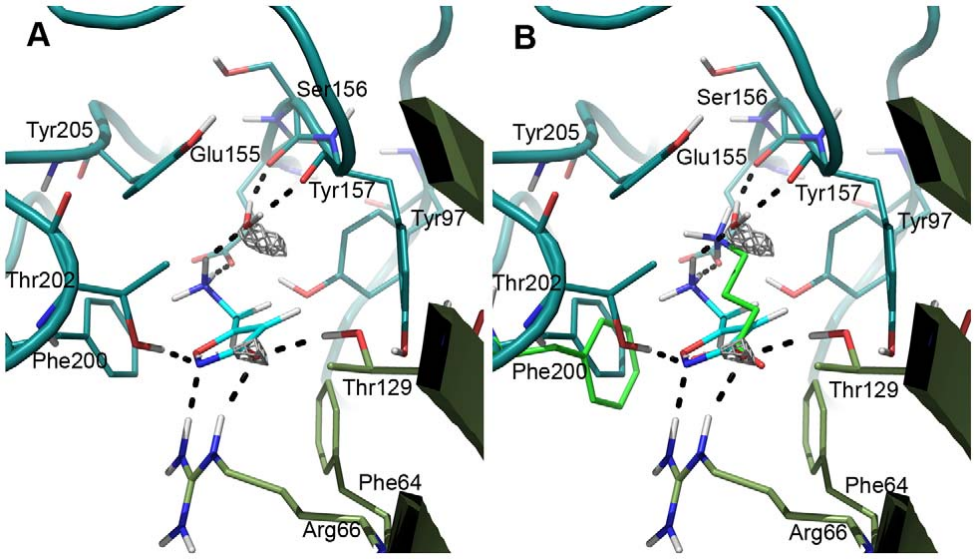
Region suited for a tightly bound water molecule identified in agonist site. A GRID calculation at the agonist binding site, using the water probe, identified two regions of strong binding interaction energy (−11 kcal/mol). One region is overlapping with the acidic moiety of agonists and the other region is situated next to the backbone of β_2_S156 and β_2_Y157 (grey mesh). The calculation was performed in absence of agonist in the binding site. In the picture, the site has been optimized for muscimol as described in the methods section. A) When a water molecule is placed between muscimol and the B-loop backbone, perfect hydrogen bonding distances are obtained, resulting in optimal interactions between the high affinity ligand muscimol and the GABA receptor. B) When also GABA is included in the site, it is obvious that the water molecule would make up for the backbone interaction that GABA is predicted to make.

The pose obtained for muscimol at first seemed erroneous, due to a slight displacement of the charged amine compared to GABA and THIP (Figure 5C). In this pose the positive charge is tucked in between the two C-loop aromatic residues β_2_Y205 and β_2_F200, however, the salt bridge to β_2_E155 is retained. The acidic moiety of the 3-hydroxy-isoxazole was perfectly placed similar to THIP and GABA. Therefore, the only difference compared to the GABA receptor interactions is a π-cation interaction with β_2_F200 instead of a backbone interaction to the B-loop. Despite numerous docking attempts and efforts to manually reposition muscimol to a pose similar to GABA and THIP, when energy minimized, the muscimol amino-methyl side chain kept “flipping” back. A dihedral drive using QM calculations revealed that the reason was a preferred torsional angle (O-C-C-N) of the muscimol amino-methyl side chain at ca. 45° (Figure 6). If both charges of muscimol were to overlay with the charges of GABA in the bound state, it would result in a torsional angle of ≥90° which would result in a conformational energy penalty of ≥3 kcal/mol (Figure 6). Such a high conformational energy is unlikely for a high affinity ligand as muscimol (K_i_ = 6 nM), which binds to GABA_A_ receptors with an even higher affinity than GABA itself (K_i_ = 18 nM) [92], [93]. The identified binding mode of muscimol depicted in Figure 5C has an O-C-C-N torsional angle of ca. 60°, which corresponds to a conformational energy penalty of ∼0.6 kcal/mol. Still, we were puzzled if no interaction with a B-loop backbone carbonyl should take place when muscimol binds. This is a generally accepted binding feature of Cys-Loop receptor agonists [26], [27], [94]. A GRID interaction energy calculation using the water probe (OH2) prior to optimization of the orthosteric receptor pocket revealed a region of high specificity for a water molecule (<−11 kcal/mol) next to the B-loop backbone carbonyls from β_2_S156 and β_2_Y157 (Figure 7A). When placing muscimol and a water molecule in the orthosteric binding site, it was found that hydrogen bonding distances were optimal and allowed muscimol a more extensive bonding pattern within the binding site than GABA. When also including the GABA binding pose, it was apparent that the GABA positive charge and the water molecule occupy the same region in the pocket (Figure 7B). We therefore propose that muscimol binds in concert with a water molecule as illustrated in Figure 7A resulting in a low conformational energy penalty and optimal interactions with the GABA_A_ orthosteric binding pocket.

### BZD binding model

**Figure 8:**
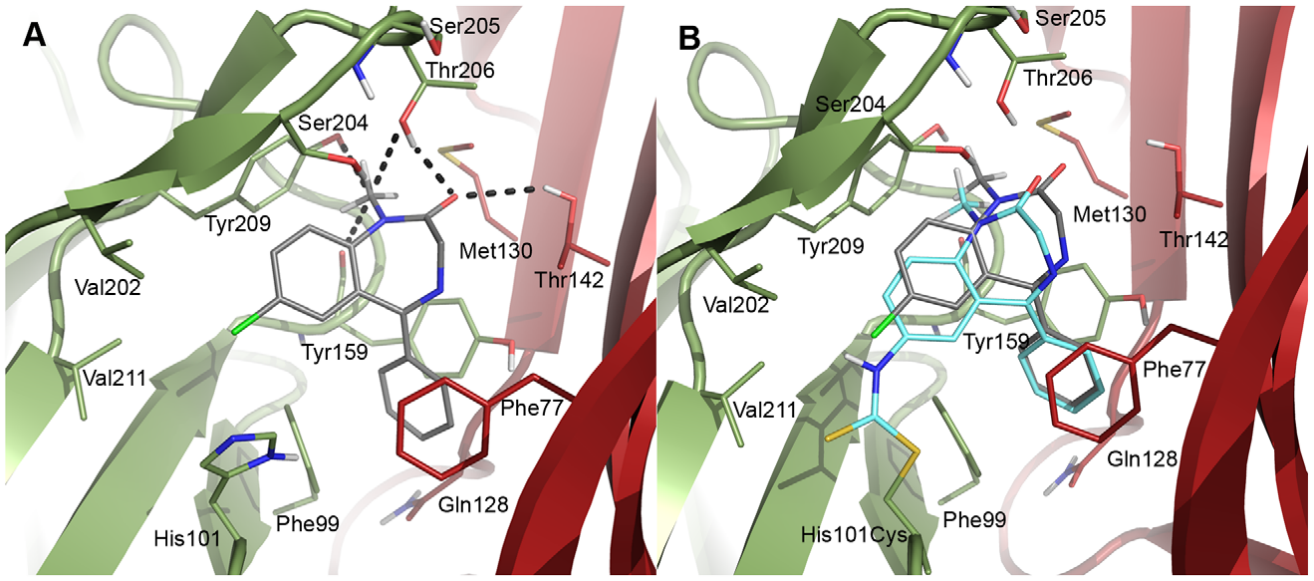
DZP binding mode. A) The assumed biologically active binding mode of DZP (gray) at the interface between the α_1_ (smudge) and γ_2_ (firebrick) subunits. In this conformation the C-3 points upwards and the pending phenyl substituent is directed inwards. B) Covalently attached DZP-NCS (cyan) overlaid with DZP (gray). Only moderate differences between the docked and the covalently attached ligands exist.

As described in the methods section DZP was manually positioned in a binding mode (Figure 8) satisfying the listed criteria deduced from experimental literature data followed by optimization of side chains in the binding pocket. The resulting model agrees to a large extent with the binding mode of DZP, recently described by Richter *et al.* [95] despite being built on different templates and is further in agreement with available mutational data (Table 2) indicating that residues α_1_F99, [67], [96] α_1_H101, [73], [97] α_1_Y159, [96], [98] α_1_Y209, [96], [98], [99] γ_2_F77, [100]–[102] and γ_2_M130 [96], [102] line the binding pocket. As was the case for the GABA othosteric binding site, the GluCl template contributes to the homology model with a higher similarity than the previously available templates. Furthermore, the ELIC X-ray structure adds information about a few BZD binding site residues (γ_2_Y58 and γ_2_F77) lacking in the GluCl structure (Figure 4B). In brief, the obtained binding mode of DZP orients the chlorine atom of DZP towards α_1_H101 and positions it underneath the C-loop pointing towards the base of the C-loop (Figure 8). The pending phenyl ring is positioned in a narrow cavity between α_1_F99 (A-loop), α_1_Y159 (B-loop), and γ_2_F77 (D-loop) in the bottom of the binding site shielded from solvent exposure. The carbonyl is positioned in a manner similar to the carboxylic acid of GABA in the orthosteric binding site. It links the two subunits at the interface by hydrogen bonds to α_1_T206 (C-loop) and γ_2_T142 (D-loop). Finally, the polarized N-methyl group is situated near the C-loop enabling polar contacts with α_1_T206, α_1_Y209 and the backbone of α_1_Y159 (Figure 8).

BZD pharmacophore models based on SAR data have predicted a lipophilic pharmacophoric feature (in the traditional BZD pharmacophore terminology named L1) to be an essential part of BZDs [71], [103]. In DZP this feature corresponds to the fused aromatic ring system, which in the model is buried beneath the C-loop and shielded from solvent by the hydrophobic residues α_1_V202, C_β_ of α_1_T206, α_1_Y209, and α_1_V211. α_1_V202 was predicted by mutational studies to line the binding site [96], [104] and α_1_T206 and α_1_Y209 are both essential for ligand binding to the BZD site [68], [69], [96], [98], [99], [102], [105]. SAR studies [71], [103] state that the pharmacophoric feature, L1, should be accommodated in a narrow cavity that does not tolerate substitution at other positions than that corresponding to C-7 in DZP (see Figure 1). Our proposed DZP binding mode comply well with this notion, since substitution at positions C-8 and C-9 would lead to steric interference with backbone atoms of the C-loop. Substitution at the C-6 position would lead to steric clashes with α_1_H101 and γ_2_F99 explaining why this is not tolerated. [71], [72] However, the cavity, in which the DZP C-7 substituent (pharmacophoric feature L2), the chlorine atom, is positioned, has to be spacious enough to accommodate larger substituents (e.g. a nitro group as seen in flunitrazepam) [72]. This is indeed the case in our model. The position of the pharmacophoric feature, L2, is further supported by studies of covalent labeling of cysteine mutants [67], [69]. In these studies, a DZP derivative, where the chlorine atom is substituted to a cysteine-reactive isothiocyanate group (DZP-NCS, Figure 1), is covalently linked to cysteine mutants of the receptor. Covalent labeling of α_1_H101C leads to a constitutively positively allosterically modulated receptor, which indicates that the ligand is covalently attached in an orientation matching the orientation of DZP in its bioactive conformation. We replicated this study *in silico* through covalent docking of DZP-NCS and obtained a pose complying with experimental data (Table 2) and resembling the otherwise established binding mode (Figure 8B) among the top scoring poses. As can be seen from the overlay of the docked DZP and the covalently attached DZP-NCS, the two ligands obtain similar binding modes, the primary difference being caused by a slight rotation of the scaffold of DZP-NCS to allow for covalent attachment of DZP-NCS. Furthermore, labeling of α_1_S205C, α_1_T206C mutants by a C-3 DZP-NCS probe strongly indicates the C-3 of DZP-NCS to point towards the tip of the C-loop [69]. Labeling with an imidazobenzodiazepine-NCS derivative of the partial negative allosteric modulator Ro15-4513, further indicates that C-7 should be oriented towards α_1_H101, α_1_N102, [67] α_1_G157, α_1_V202, and α_1_V211, [68] also correlating with our model.

Available SAR data for BZDs indicate that the pending phenyl ring (pharmacophoric feature L3) is positioned in a narrow cavity [71], [72]. Substitution at the ring is only tolerated in the 2′-position, whereas substitution in positions 3′ and 4′ are poorly tolerated. [71], [72] Our model agrees with these observations, as the pending phenyl ring is positioned in an aromatic box formed by α_1_F99, α_1_H101, α_1_Y159, γ_2_F99, and γ_2_N128. In addition to satisfying ligand requirements of aromatic burial, the described position of the pending phenyl ring also serves as a strong contact point between the α_1_ and the γ_2_ subunits.

The remaining lipophilic pharmacophoric features L3 (corresponding to the phenyl group in DZP) and L_DI_ of the traditional benzodiazepine pharmacophore models [14], [71], [103] are situated near residues α_1_V202 and α_1_V211, and γ_2_A79, γ_2_L140, γ_2_Y58, γ_2_Q56, respectively. The latter feature is not occupied by DZP but is important for binding and function of other BZD binding site ligands, e.g. indol-3-yl-glyoxylamides [106].

An additional point of inter subunit contact is the carbonyl group of DZP. This carbonyl is positioned under the tip of the C-loop and is in an optimal position to form hydrogen bonds across the interface through α_1_T206 and γ_2_T142, similar to how the GABA carboxylate binds to β_2_T202 and α_1_T129 in the orthosteric binding site. Indeed, mutation of α_1_T206 has been identified in several studies to severely affect binding affinity of BZDs (Table 2).

Further interactions between DZP and the receptor model are formed by the polarized *N*-methyl group. Due to the position of the methyl group on a nitrogen atom, the hydrogen atoms are more polarized than they would be if the methyl group was attached to an aliphatic carbon, thereby being able to form polar contacts with the receptor. This has been seen previously in AChBP X-ray structures [94]. In our model these hydrogen atoms are positioned in the negatively charged electrostatic field of the backbone carbonyl oxygen of α_1_Y159 and the hydroxyl groups of α_1_T206 and α_1_Y209. This also explains the beneficial effect of including the *N*-methyl substituent in BZDs. [72]

Interestingly, when comparing the validated binding modes of GABA and DZP in their respective binding sites it is intriguing to see that corresponding residues, in particular β_2_T202/α_1_T206, β_2_Y205/α_1_Y209, β_2_Y97/α_1_F99, β_2_Y157/α_1_Y159, α_1_T129/γ_2_T142 and α_1_F64/γ_2_F77 are responsible for contacts between receptor and ligands and that both GABA and DZP in a similar manner bridge neighboring subunits. We have recently shown that a third binding site exists in the α_4_α_4_ interface of the so-called low sensitivity nAChR (α_4_)_3_(β_2_)_2_ and hypothesized that this could be the nicotinic pendant of the BZD binding site [107]. The structural model presented here may help to design experiments to investigate this hypothesis and thus contribute to the ongoing debate of the mechanism of action of BZDs [96], [98], [108]–[111].

## Conclusions

The model and subsequent validation by available experimental data shows that reliable GABA_A_R models can be obtained using novel full length receptor templates. In addition to serving as a model of how agonists and modulators may bind to the GABA_A_R, the model may help to guide mutational studies unraveling the mechanism by which agonists, BZDs and other allosteric modulators work.

Compared to earlier templates used for homology modeling of the GABA_A_Rs the emergence of the X-ray structure of the GluCl ion channel has significantly increased the insight into the architecture of anionic Cys-Loop receptors. With the new templates, sequence identities with respect to GABA sequences are as high as 36% and even up to 48% if narrowing the focus to the agonist binding site. Hence, the information on side chain conformations in the binding site of GABA_A_R/anion channels of the Cys-Loop receptor family has now improved considerably. Previously published homology models have mainly been modeled with AChBP as template. With sequence identities as low as 19% combined with the lack of a TMD as is the case when using AChBPs as templates, homology models based on the GluCl structure represents a big step forward.

Based on homology modeling, advanced docking methods, QM calculations and a vast amount of collected experimental data, we have identified binding hypotheses for GABA, muscimol, THIP and diazepam and optimized the binding sites accordingly. Our GABA_A_R model is modeled in the open state according to the GluCl glutamate bound structure and is intended for creating binding hypotheses of agonists or BZD site modulators. The model is made available in Model S1.

## Supporting Information

Model S1 **The GABA_A_R model described in this paper.**
(ZIP)

Figure S1 **Calculations of binding site sequence identities.**
(PDF)

Figure S2 **RMSD plot and details of a 48 ns molecular dynamics simulation.**
(PDF)

## References

[1] Johnston GA (2005) GABA(A) receptor channel pharmacology. Curr Pharm Des 11: 1867–1885. 15974965 10.2174/1381612054021024

[2] Johnston GA, Curtis DR, De Groat WC, Duggan AW (1968) Central actions of ibotenic acid and muscimol. Biochem Pharmacol 17: 2488–2489. 5752907 10.1016/0006-2952(68)90141-x

[3] Ebert B, Frolund B, Diemer NH, Krogsgaard-Larsen P (1999) Equilibrium binding characteristics of [3H]thiomuscimol. Neurochem Int 34: 427–434. 10397371 10.1016/s0197-0186(99)00038-8

[4] Krogsgaard-Larsen P, Johnston GA, Lodge D, Curtis DR (1977) A new class of GABA agonist. Nature 268: 53–55. 196200 10.1038/268053a0

[5] Frolund B, Kristiansen U, Brehm L, Hansen AB, Krogsgaard-Larsen P, et al (1995) Partial GABAA receptor agonists. Synthesis and in vitro pharmacology of a series of nonannulated analogs of 4,5,6,7-tetrahydroisoxazolo[5,4-c]pyridin-3-ol. J Med Chem 38: 3287–3296. 7650683 10.1021/jm00017a014

[6] McKernan RM, Rosahl TW, Reynolds DS, Sur C, Wafford KA, et al (2000) Sedative but not anxiolytic properties of benzodiazepines are mediated by the GABA_A_ receptor α_1_ subtype. Nat Neurosci 3: 587–592. 10816315 10.1038/75761

[7] Rosahl TW, Sur C, Reynolds DS, Collinson N, Macauley A, et al (2000) Towards an understanding of the role or the GABAergic system in anxiety, learning and memory. European Journal of Neuroscience 12: 514–514.

[8] Grant JA, Bonnick T, Gossell-Williams M, Clayton T, Cook JM, et al (2010) Synthesis, pharmacological studies and molecular modeling of some tetracyclic 1,3-diazepinium chlorides. Bioorg Med Chem 18: 909–921. 19962901 10.1016/j.bmc.2009.11.032

[9] He X, Huang Q, Ma C, Yu S, McKernan R, et al (2000) Pharmacophore/receptor models for GABA_A_/BzR α2β3γ2, α3β3γ2 and α4β3γ2 recombinant subtypes. Included volume analysis and comparison to α1β3γ2, α5β3γ2, and α6β3γ2 subtypes. Drug Des Discov 17: 131–171. 11045902

[10] Huang Q, Cox ED, Gan T, Ma C, Bennett DW, et al (1999) Studies of molecular pharmacophore/receptor models for GABAA/benzodiazepine receptor subtypes: binding affinities of substituted beta-carbolines at recombinant alpha x beta 3 gamma 2 subtypes and quantitative structure-activity relationship studies via a comparative molecular field analysis. Drug Des Discov 16: 55–76. 10466057

[11] Ghoshal N, Vijayan RSK (2010) Pharmacophore models for GABA_A_ modulators: implications in CNS drug discovery. Exp Opin Drug Discov 5: 441–460. 10.1517/17460441003789363 22823129

[12] Frolund B, Tagmose L, Liljefors T, Stensbol TB, Engblom C, et al (2000) A novel class of potent 3-isoxazolol GABA(A) antagonists: design, synthesis, and pharmacology. J Med Chem 43: 4930–4933. 11150163 10.1021/jm000371q

[13] Krogsgaard-Larsen P, Frølund B, Liljefors T (2002) Specific GABA(A) agonists and partial agonists. Chem Rec 2: 419–430. 12469353 10.1002/tcr.10040

[14] Clayton T, Chen JL, Ernst M, Richter L, Cromer BA, et al (2007) An updated unified pharmacophore model of the benzodiazepine binding site on gamma-aminobutyric acid(a) receptors: correlation with comparative models. Curr Med Chem 14: 2755–2775. 18045122 10.2174/092986707782360097

[15] Berezhnoy D, Gibbs TT, Farb DH (2009) Docking of 1,4-benzodiazepines in the α_1_/γ_2_ GABA_A_ receptor modulator site. Mol Pharmacol 76: 440–450. 19483108 10.1124/mol.109.054650 PMC2713131

[16] Ci SQ, Ren TR, Ma CX, Su ZG (2007) Modeling of αk/γ2 (k = 1, 2, 3 and 5) interface of GABA_A_ receptor and docking studies with zolpidem: implications for selectivity. J Mol Graph Model 26: 537–545. 17451983 10.1016/j.jmgm.2007.03.007

[17] Cromer BA, Morton CJ, Parker MW (2002) Anxiety over GABA_A_ receptor structure relieved by AChBP. Trends Biochem Sci 27: 280–287. 12069787 10.1016/s0968-0004(02)02092-3

[18] Sancar F, Ericksen SS, Kucken AM, Teissere JA, Czajkowski C (2007) Structural determinants for high-affinity zolpidem binding to GABA-A receptors. Mol Pharmacol 71: 38–46. 17012619 10.1124/mol.106.029595

[19] Gharaghani S, Khayamian T, Keshavarz F (2011) A structure-based QSAR and docking study on imidazo[1,5-a][1,2,4]-triazolo[1,5-d][1,4,]benzodiazepines as Selective GABA(A) alpha5 inverse agonists. Chemical biology & drug design 78: 612–621. 21756285 10.1111/j.1747-0285.2011.01183.x

[20] Ernst M, Brauchart D, Boresch S, Sieghart W (2003) Comparative modeling of GABA_A_ receptors: limits, insights, future developments. Neuroscience 119: 933–943. 12831854 10.1016/s0306-4522(03)00288-4

[21] Sawyer GW, Chiara DC, Olsen RW, Cohen JB (2002) Identification of the bovine γ-aminobutyric acid type A receptor α subunit residues photolabeled by the imidazobenzodiazepine [^3^H]Ro15-4513. J Biol Chem 277: 50036–50045. 12388542 10.1074/jbc.M209281200

[22] Ci S, Ren T, Su Z (2008) Investigating the putative binding-mode of GABA and diazepam within GABA A receptor using molecular modeling. Protein J 27: 71–78. 17805947 10.1007/s10930-007-9109-9

[23] Law RJ, Lightstone FC (2009) Modeling neuronal nicotinic and GABA receptors: important interface salt-links and protein dynamics. Biophys J 97: 1586–1594. 19751663 10.1016/j.bpj.2009.06.044 PMC2749782

[24] Cheng J, Ju XL (2010) Homology modeling and atomic level binding study of GABA(A) receptor with novel enaminone amides. Eur J Med Chem 45: 3595–3600. 20684859 10.1016/j.ejmech.2010.05.004

[25] O'Mara M, Cromer B, Parker M, Chung SH (2005) Homology model of the GABAA receptor examined using Brownian dynamics. Biophys J 88: 3286–3299. 15749776 10.1529/biophysj.104.051664 PMC1305477

[26] Brejc K, van Dijk WJ, Klaassen RV, Schuurmans M, van Der Oost J, et al (2001) Crystal structure of an ACh-binding protein reveals the ligand-binding domain of nicotinic receptors. Nature 411: 269–276. 11357122 10.1038/35077011

[27] Hansen SB, Sulzenbacher G, Huxford T, Marchot P, Taylor P, et al (2005) Structures of Aplysia AChBP complexes with nicotinic agonists and antagonists reveal distinctive binding interfaces and conformations. Embo J 24: 3635–3646. 16193063 10.1038/sj.emboj.7600828 PMC1276711

[28] Chang YC, Wu W, Zhang JL, Huang Y (2009) Allosteric activation mechanism of the cys-loop receptors. Acta Pharmacol Sin 30: 663–672. 19444220 10.1038/aps.2009.51 PMC4002373

[29] Dutertre S, Ulens C, Buttner R, Fish A, van Elk R, et al (2007) AChBP-targeted alpha-conotoxin correlates distinct binding orientations with nAChR subtype selectivity. The EMBO journal 26: 3858–3867. 17660751 10.1038/sj.emboj.7601785 PMC1952216

[30] Unwin N (2005) Refined structure of the nicotinic acetylcholine receptor at 4 Å resolution. J Mol Biol 346: 967–989. 15701510 10.1016/j.jmb.2004.12.031

[31] Bisson WH, Westera G, Schubiger PA, Scapozza L (2008) Homology modeling and dynamics of the extracellular domain of rat and human neuronal nicotinic acetylcholine receptor subtypes alpha4beta2 and alpha7. Journal of Molecular Modeling 14: 891–899. 18607650 10.1007/s00894-008-0340-x

[32] Harpsoe K, Ahring PK, Christensen JK, Jensen ML, Peters D, et al (2011) Unraveling the high- and low-sensitivity agonist responses of nicotinic acetylcholine receptors. The Journal of neuroscience: the official journal of the Society for Neuroscience 31: 10759–10766. 21795528 10.1523/JNEUROSCI.1509-11.2011 PMC6623092

[33] Celie PH, van Rossum-Fikkert SE, van Dijk WJ, Brejc K, Smit AB, et al (2004) Nicotine and carbamylcholine binding to nicotinic acetylcholine receptors as studied in AChBP crystal structures. Neuron 41: 907–914. 15046723 10.1016/s0896-6273(04)00115-1

[34] Dellisanti CD, Yao Y, Stroud JC, Wang ZZ, Chen L (2007) Crystal structure of the extracellular domain of nAChR alpha1 bound to alpha-bungarotoxin at 1.94 A resolution. Nat Neurosci 10: 953–962. 17643119 10.1038/nn1942

[35] Bocquet N, Nury H, Baaden M, Le Poupon C, Changeux JP, et al (2009) X-ray structure of a pentameric ligand-gated ion channel in an apparently open conformation. Nature 457: 111–114. 18987633 10.1038/nature07462

[36] Hilf RJC, Dutzler R (2008) X-ray structure of a prokaryotic pentameric ligand-gated ion channel. Nature 452: 375–379. 18322461 10.1038/nature06717

[37] Sander T, Frolund B, Bruun AT, Ivanov I, McCammon JA, et al (2011) New insights into the GABA(A) receptor structure and orthosteric ligand binding: receptor modeling guided by experimental data. Proteins 79: 1458–1477. 21365676 10.1002/prot.22975 PMC3076690

[38] Hibbs RE, Gouaux E (2011) Principles of activation and permeation in an anion-selective Cys-loop receptor. Nature 474: 54–60. 21572436 10.1038/nature10139 PMC3160419

[39] Frolund B, Jorgensen AT, Tagmose L, Stensbol TB, Vestergaard HT, et al (2002) Novel class of potent 4-arylalkyl substituted 3-isoxazolol GABA(A) antagonists: synthesis, pharmacology, and molecular modeling. J Med Chem 45: 2454–2468. 12036354 10.1021/jm020027o

[40] Hilf RJ, Dutzler R (2008) X-ray structure of a prokaryotic pentameric ligand-gated ion channel. Nature 452: 375–379. 18322461 10.1038/nature06717

[41] Berman HM, Westbrook J, Feng Z, Gilliland G, Bhat TN, et al (2000) The Protein Data Bank. Nucleic Acids Res 28: 235–242. 10592235 10.1093/nar/28.1.235 PMC102472

[42] Larkin MA, Blackshields G, Brown NP, Chenna R, McGettigan PA, et al (2007) Clustal W and Clustal X version 2.0. Bioinformatics 23: 2947–2948. 17846036 10.1093/bioinformatics/btm404

[43] Celie PH, Klaassen RV, van Rossum-Fikkert SE, van Elk R, van Nierop P, et al (2005) Crystal structure of acetylcholine-binding protein from Bulinus truncatus reveals the conserved structural scaffold and sites of variation in nicotinic acetylcholine receptors. J Biol Chem 280: 26457–26466. 15899893 10.1074/jbc.M414476200

[44] Sali A, Blundell TL (1993) Comparative protein modelling by satisfaction of spatial restraints. J Mol Biol 234: 779–815. 8254673 10.1006/jmbi.1993.1626

[45] Sippl MJ (1993) Recognition of errors in three-dimensional structures of proteins. Proteins 17: 355–362. 8108378 10.1002/prot.340170404

[46] Kaminski GA, Friesner RA, Tirado-Rives J, Jorgensen WL (2001) Evaluation and reparametrization of the OPLS-AA force field for proteins via comparison with accurate quantum chemical calculations on peptides. Journal of Physical Chemistry B 105: 6474–6487.

[47] Jorgensen WL, Maxwell DS, TiradoRives J (1996) Development and testing of the OPLS all-atom force field on conformational energetics and properties of organic liquids. Journal of the American Chemical Society 118: 11225–11236.

[48] Schrödinger L (2010) Maestro. 9.1 ed. New York, NY, USA.

[49] Olsson MHM, Sondergaard CR, Rostkowski M, Jensen JH (2011) PROPKA3: Consistent Treatment of Internal and Surface Residues in Empirical pK(a) Predictions. Journal of Chemical Theory and Computation 7: 525–537. 26596171 10.1021/ct100578z

[50] Jensen JH, Bas DC, Rogers DM (2008) Very fast prediction and rationalization of pK(a) values for protein-ligand complexes. Proteins-Structure Function and Bioinformatics 73: 765–783. 10.1002/prot.22102 18498103

[51] Li H, Robertson AD, Jensen JH (2005) Very fast empirical prediction and rationalization of protein pK(a) values. Proteins-Structure Function and Bioinformatics 61: 704–721. 10.1002/prot.20660 16231289

[52] Schrödinger L (2010) Prime. 2.2 ed. New York, NY, USA.

[53] Maestro v. 9.2, Schrödinger, LLC, New York, NY, 2011.

[54] MacroModel v. 9.9, Schrödinger, LLC, New York, NY, 2011.

[55] Sherman W, Beard HS, Farid R (2006) Use of an induced fit receptor structure in virtual screening. Chem Biol Drug Des 67: 83–84. 16492153 10.1111/j.1747-0285.2005.00327.x

[56] Sherman W, Day T, Jacobson MP, Friesner RA, Farid R (2006) Novel procedure for modeling ligand/receptor induced fit effects. J Med Chem 49: 534–553. 16420040 10.1021/jm050540c

[57] Friesner RA, Murphy RB, Repasky MP, Frye LL, Greenwood JR, et al (2006) Extra precision glide: docking and scoring incorporating a model of hydrophobic enclosure for protein-ligand complexes. J Med Chem 49: 6177–6196. 17034125 10.1021/jm051256o

[58] Goodford PJ (1985) A computational procedure for determining energetically favorable binding sites on biologically important macromolecules. J Med Chem 28: 849–857. 3892003 10.1021/jm00145a002

[59] Molecular Discovery L (2005) GRID 22. Pinner, Middlesex, UK.

[60] Jaguar v. 7.8, Schrödinger, LLC, New York, NY, 2011.

[61] Tannor DJ, Marten B, Murphy R, Friesner RA, Sitkoff D, et al (1994) Accurate First Principles Calculation of Molecular Charge Distributions and Solvation Energies from Ab Initio Quantum Mechanics and Continuum Dielectic Theory. J Am Chem Soc 116: 11875–11882.

[62] Young R, Glennon RA, Dewey WL (1984) Stereoselective stimulus effects of 3-methylflunitrazepam and pentobarbital. Life Sci 34: 1977–1983. 6328149 10.1016/0024-3205(84)90129-2

[63] Fellegvari I, Visy J, Valko K, Lang T, Simonyi M (1989) Investigation of Conformational Diastereomers of 2,3-Benzodiazepines by High-Performance Liquid-Chromatography. J Liq Chromatogr 12: 2719–2732.

[64] Simonyi M (1990) Chiral recognition by central benzodiazepine receptors. Acta Pharm Nord 2: 145–154. 2166530

[65] Maksay G, Tegyey Z, Simonyi M (1991) Central benzodiazepine receptors: in vitro efficacies and potencies of 3-substituted 1,4-benzodiazepine stereoisomers. Mol Pharmacol 39: 725–732. 1646947

[66] Simonyi M, Maksay G (1990) Conformational Recognition by Central Benzodiazepine Receptors. Bioorg Chem 18: 1–12.

[67] Tan KR, Baur R, Gonthier A, Goeldner M, Sigel E (2007) Two neighboring residues of loop A of the alpha1 subunit point towards the benzodiazepine binding site of GABAA receptors. FEBS Lett 581: 4718–4722. 17854801 10.1016/j.febslet.2007.08.068

[68] Tan KR, Gonthier A, Baur R, Ernst M, Goeldner M, et al (2007) Proximity-accelerated chemical coupling reaction in the benzodiazepine-binding site of gamma-aminobutyric acid type A receptors: superposition of different allosteric modulators. J Biol Chem 282: 26316–26325. 17626010 10.1074/jbc.M702153200

[69] Tan KR, Baur R, Charon S, Goeldner M, Sigel E (2009) Relative positioning of diazepam in the benzodiazepine-binding-pocket of GABA receptors. J Neurochem 111: 1264–1273. 19804380 10.1111/j.1471-4159.2009.06419.x

[70] Sigel E, Stephenson FA, Mamalaki C, Barnard EA (1983) A gamma-aminobutyric acid/benzodiazepine receptor complex of bovine cerebral cortex. J Biol Chem 258: 6965–6971. 6304068

[71] Zhang W, Koehler KF, Zhang P, Cook JM (1995) Development of a comprehensive pharmacophore model for the benzodiazepine receptor. Drug Des Discov 12: 193–248. 7662830

[72] Sternbach LH (1971) 1,4-benzodiazepines. Chemistry and some aspects of the structure-activity relationship. Angew Chem Int Ed Engl 10: 34–43. 4993720 10.1002/anie.197100341

[73] Berezhnoy D, Nyfeler Y, Gonthier A, Schwob H, Goeldner M, et al (2004) On the benzodiazepine binding pocket in GABAA receptors. J Biol Chem 279: 3160–3168. 14612433 10.1074/jbc.M311371200

[74] Berezhnoy D, Baur R, Gonthier A, Foucaud B, Goeldner M, et al (2005) Conformational changes at benzodiazepine binding sites of GABA(A) receptors detected with a novel technique. J Neurochem 92: 859–866. 15686488 10.1111/j.1471-4159.2004.02913.x

[75] Laskowski RA, MacArthur MW, Moss DS, Thornton JM (1993) PROCHECK - a program to check the stereochemical quality of protein structures. J App Cryst 26: 283–291.

[76] Newell JG, McDevitt RA, Czajkowski C (2004) Mutation of glutamate 155 of the GABAA receptor beta2 subunit produces a spontaneously open channel: a trigger for channel activation. J Neurosci 24: 11226–11235. 15601928 10.1523/JNEUROSCI.3746-04.2004 PMC6730373

[77] Thompson AJ, Alqazzaz M, Ulens C, Lummis SC (2012) The pharmacological profile of ELIC, a prokaryotic GABA-gated receptor. Neuropharmacology 63: 761–767. 22677470 10.1016/j.neuropharm.2012.05.027 PMC3430861

[78] Wagner DA, Czajkowski C, Jones MV (2004) An arginine involved in GABA binding and unbinding but not gating of the GABA(A) receptor. J Neurosci 24: 2733–2741. 15028766 10.1523/JNEUROSCI.4316-03.2004 PMC6729509

[79] Steward EG, Player RB, Warner D (1973) the Crystal Structure of gamma-Aminobutyric Acid Hydrochloride: A refinement. Acta Cryst B 29: 2825–2826.

[80] Tomita K (1965) Jap J Brain Physiol. 61: 1–4.

[81] Amin J, Weiss DS (1993) GABAA receptor needs two homologous domains of the beta-subunit for activation by GABA but not by pentobarbital. Nature 366: 565–569. 7504783 10.1038/366565a0

[82] Wagner DA, Czajkowski C (2001) Structure and dynamics of the GABA binding pocket: A narrowing cleft that constricts during activation. J Neurosci 21: 67–74. 11150321 10.1523/JNEUROSCI.21-01-00067.2001 PMC6762441

[83] Gynther BD, Curtis DR (1986) Pyridazinyl-GABA derivatives as GABA and glycine antagonists in the spinal cord of the cat. Neurosci Lett 68: 211–215. 3018632 10.1016/0304-3940(86)90144-8

[84] Boileau AJ, Evers AR, Davis AF, Czajkowski C (1999) Mapping the agonist binding site of the GABAA receptor: evidence for a beta-strand. J Neurosci 19: 4847–4854. 10366619 10.1523/JNEUROSCI.19-12-04847.1999 PMC6782682

[85] Jansen M, Rabe H, Strehle A, Dieler S, Debus F, et al (2008) Synthesis of GABAA receptor agonists and evaluation of their alpha-subunit selectivity and orientation in the GABA binding site. J Med Chem 51: 4430–4448. 18651727 10.1021/jm701562x PMC2566937

[86] Holden JH, Czajkowski C (2002) Different residues in the GABA(A) receptor alpha 1T60-alpha 1K70 region mediate GABA and SR-95531 actions. J Biol Chem 277: 18785–18792. 11896052 10.1074/jbc.M111778200

[87] Harrison NJ, Lummis SC (2006) Molecular modeling of the GABA(C) receptor ligand-binding domain. J Mol Model 12: 317–324. 16249935 10.1007/s00894-005-0034-6

[88] Cromer BA, Morton CJ, Parker MW (2002) Anxiety over GABA(A) receptor structure relieved by AChBP. Trends Biochem Sci 27: 280–287. 12069787 10.1016/s0968-0004(02)02092-3

[89] Kloda JH, Czajkowski C (2007) Agonist-, antagonist-, and benzodiazepine-induced structural changes in the alpha1 Met113-Leu132 region of the GABAA receptor. Mol Pharmacol 71: 483–493. 17108261 10.1124/mol.106.028662

[90] Hibbs RE, Sulzenbacher G, Shi J, Talley TT, Conrod S, et al (2009) Structural determinants for interaction of partial agonists with acetylcholine binding protein and neuronal alpha7 nicotinic acetylcholine receptor. Embo J 28: 3040–3051. 19696737 10.1038/emboj.2009.227 PMC2760105

[91] Padgett CL, Hanek AP, Lester HA, Dougherty DA, Lummis SC (2007) Unnatural amino acid mutagenesis of the GABA(A) receptor binding site residues reveals a novel cation-pi interaction between GABA and beta 2Tyr97. J Neurosci 27: 886–892. 17251430 10.1523/JNEUROSCI.4791-06.2007 PMC2649369

[92] Boström J, Norrby PO, Liljefors T (1998) Conformational energy penalties of protein-bound ligands. J Comput Aided Mol Des 12: 383–396. 9777496 10.1023/a:1008007507641

[93] Madsen C, Jensen AA, Liljefors T, Kristiansen U, Nielsen B, et al (2007) 5-Substituted imidazole-4-acetic acid analogues: synthesis, modeling, and pharmacological characterization of a series of novel gamma-aminobutyric acid(C) receptor agonists. J Med Chem 50: 4147–4161. 17655213 10.1021/jm070447j

[94] Rohde LA, Ahring PK, Jensen ML, Nielsen EO, Peters D, et al (2012) Intersubunit bridge formation governs agonist efficacy at nicotinic acetylcholine alpha4beta2 receptors: unique role of halogen bonding revealed. J Biol Chem 287: 4248–4259. 22170047 10.1074/jbc.M111.292243 PMC3281707

[95] Richter L, de Graaf C, Sieghart W, Varagic Z, Morzinger M, et al (2012) Diazepam-bound GABA(A) receptor models identify new benzodiazepine binding-site ligands. Nat Chem Biol 8: 455–464. 22446838 10.1038/nchembio.917 PMC3368153

[96] Hanson SM, Morlock EV, Satyshur KA, Czajkowski C (2008) Structural requirements for eszopiclone and zolpidem binding to the gamma-aminobutyric acid type-A (GABAA) receptor are different. J Med Chem 51: 7243–7252. 18973287 10.1021/jm800889m PMC2645942

[97] Davies M, Bateson AN, Dunn SM (1998) Structural requirements for ligand interactions at the benzodiazepine recognition site of the GABA(A) receptor. J Neurochem 70: 2188–2194. 9572307 10.1046/j.1471-4159.1998.70052188.x

[98] Amin J, Brooks-Kayal A, Weiss DS (1997) Two tyrosine residues on the alpha subunit are crucial for benzodiazepine binding and allosteric modulation of gamma-aminobutyric acidA receptors. Mol Pharmacol 51: 833–841. 9145922 10.1124/mol.51.5.833

[99] Buhr A, Schaerer MT, Baur R, Sigel E (1997) Residues at positions 206 and 209 of the alpha1 subunit of gamma-aminobutyric AcidA receptors influence affinities for benzodiazepine binding site ligands. Mol Pharmacol 52: 676–682. 9380031 10.1124/mol.52.4.676

[100] Buhr A, Baur R, Sigel E (1997) Subtle changes in residue 77 of the gamma subunit of alpha1beta2gamma2 GABAA receptors drastically alter the affinity for ligands of the benzodiazepine binding site. J Biol Chem 272: 11799–11804. 9115236 10.1074/jbc.272.18.11799

[101] Wingrove PB, Thompson SA, Wafford KA, Whiting PJ (1997) Key amino acids in the gamma subunit of the gamma-aminobutyric acidA receptor that determine ligand binding and modulation at the benzodiazepine site. Mol Pharmacol 52: 874–881. 9351978 10.1124/mol.52.5.874

[102] Sigel E, Schaerer MT, Buhr A, Baur R (1998) The benzodiazepine binding pocket of recombinant alpha1beta2gamma2 gamma-aminobutyric acidA receptors: relative orientation of ligands and amino acid side chains. Mol Pharmacol 54: 1097–1105. 9855639 10.1124/mol.54.6.1097

[103] Diaz-Arauzo H, Koehler KF, Hagen TJ, Cook JM (1991) Synthetic and computer assisted analysis of the pharmacophore for agonists at benzodiazepine receptors. Life Sci 49: 207–216. 1648158 10.1016/0024-3205(91)90005-v

[104] Buhr A, Baur R, Malherbe P, Sigel E (1996) Point mutations of the alpha 1 beta 2 gamma 2 gamma-aminobutyric acid(A) receptor affecting modulation of the channel by ligands of the benzodiazepine binding site. Mol Pharmacol 49: 1080–1084. 8649346

[105] Schaerer MT, Buhr A, Baur R, Sigel E (1998) Amino acid residue 200 on the alpha1 subunit of GABA(A) receptors affects the interaction with selected benzodiazepine binding site ligands. Eur J Pharmacol 354: 283–287. 9754930 10.1016/s0014-2999(98)00456-7

[106] Taliani S, Cosimelli B, Da Settimo F, Marini AM, La Motta C, et al (2009) Identification of anxiolytic/nonsedative agents among indol-3-ylglyoxylamides acting as functionally selective agonists at the gamma-aminobutyric acid-A (GABAA) alpha2 benzodiazepine receptor. J Med Chem 52: 3723–3734. 19469479 10.1021/jm9001154

[107] Harpsoe K, Ahring PK, Christensen JK, Jensen ML, Peters D, et al (2011) Unraveling the high- and low-sensitivity agonist responses of nicotinic acetylcholine receptors. J Neurosci 31: 10759–10766. 21795528 10.1523/JNEUROSCI.1509-11.2011 PMC6623092

[108] Campo-Soria C, Chang Y, Weiss DS (2006) Mechanism of action of benzodiazepines on GABAA receptors. Br J Pharmacol 148: 984–990. 16783415 10.1038/sj.bjp.0706796 PMC1751932

[109] Walters RJ, Hadley SH, Morris KD, Amin J (2000) Benzodiazepines act on GABAA receptors via two distinct and separable mechanisms. Nat Neurosci 3: 1274–1281. 11100148 10.1038/81800

[110] Sharkey LM, Czajkowski C (2008) Individually monitoring ligand-induced changes in the structure of the GABAA receptor at benzodiazepine binding site and non-binding-site interfaces. Mol Pharmacol 74: 203–312. 18424553 10.1124/mol.108.044891 PMC2552402

[111] Morlock EV, Czajkowski C (2011) Different residues in the GABAA receptor benzodiazepine binding pocket mediate benzodiazepine efficacy and binding. Mol Pharmacol 80: 14–22. 21447642 10.1124/mol.110.069542 PMC3127544

[112] Venkatachalan SP, Czajkowski C (2008) A conserved salt bridge critical for GABA(A) receptor function and loop C dynamics. Proc Natl Acad Sci U S A 105: 13604–13609. 18757734 10.1073/pnas.0801854105 PMC2533236

[113] Sigel E, Baur R, Kellenberger S, Malherbe P (1992) Point mutations affecting antagonist affinity and agonist dependent gating of GABAA receptor channels. Embo J 11: 2017–2023. 1376242 10.1002/j.1460-2075.1992.tb05258.x PMC556666

[114] Westh-Hansen SE, Witt MR, Dekermendjian K, Liljefors T, Rasmussen PB, et al (1999) Arginine residue 120 of the human GABAA receptor alpha 1, subunit is essential for GABA binding and chloride ion current gating. Neuroreport 10: 2417–2421. 10439474 10.1097/00001756-199908020-00036

[115] Boileau AJ, Newell JG, Czajkowski C (2002) GABA(A) receptor beta 2 Tyr97 and Leu99 line the GABA-binding site. Insights into mechanisms of agonist and antagonist actions. J Biol Chem 277: 2931–2937. 11711541 10.1074/jbc.M109334200

[116] Wingrove PB, Safo P, Wheat L, Thompson SA, Wafford KA, et al (2002) Mechanism of alpha-subunit selectivity of benzodiazepine pharmacology at gamma-aminobutyric acid type A receptors. Eur J Pharmacol 437: 31–39. 11864636 10.1016/s0014-2999(02)01279-7

