# Supplementary material for: A Unified Model of the GABAA Receptor Comprising Agonist and Benzodiazepine Binding Sites
Source: PLoS One. 2013 Jan 7;8(1):e52323. doi: 10.1371/journal.pone.0052323 (PMC3538749; doi:10.1371/journal.pone.0052323)
Supplement: Figure S1 — Calculations of binding site sequence identities. (PDF) [file pone.0052323.s002.pdf]

Supporting Information – Figure S1

GABA site

Residues having at least one atom within 8 Å of Glu in the GluCl structure

```
       *         ** **     *   * *              *    * * *    **  *
GluCl  D.F.P .. ASYAYT .. SVTNTGIYSC N.L.R .. TLR .. NVL.R .. LYSVR .. Q.K
GABA   D.Y.L .. ESYGYT .. VVFSTGSYPR D.F.T .. FRR .. NKL.R .. LYTMR .. V.A
GABA   beta subunit                  alpha subunit
```

15 conserved residues (*) (~39%)

42 residues in total

Residues having at least one atom within 8 Å of Glu in the GluCl structure **AND** pointing towards the binding site

```
Res.#  89       149    193           33       54     107      119      169
       *        .**        **          .        *    * * *    *   *
GluCl  D.F.P .. ASY .. S.T.TG        N.L.R .. T.R .. N.L.R .. L.S.R .. Q.K
ELIC            EPF                    F.N .. Y
GABA   D.Y.L .. ESY .. V.F.TG        D.F.T .. F.R .. N.L.R .. L.T.R .. V.A
GABA   beta subunit                  alpha subunit
```

11 conserved residues to GluCl alone (*) (~48%)

13 conserved residues to the sum of the templates (* and .) (~57%)

23 residues in total

BZD site

Residues having at least one atom within 8 Å of Glu in the GluCl structure **AND** pointing towards the binding site

```
Res.#  89       149    193           33       54     107      119      169
       * *       **        **            .      *    *   *    *   *
GluCl  D.F.P .. ASY .. S.T.TG        N.L.R .. T.R .. N.L.R .. L.S.R .. Q.K
ELIC            EPF                    F.N .. Y
GABA   D.F.H .. GSY .. V.S.TG        D.Y.N .. F.A .. N.M.R .. L.T.R .. E.G
GABA   alpha subunit                 gamma subunit
```

10 conserved residues to GluCl alone (*) (~43%)

11 conserved residues to the sum of the templates (* and .) (~48%)

23 residues in total

Figure S1. Calculated sequence identities for GABA and BZD binding sites with respect to the templates used in homology modeling. Residue numbers are according to GluCl. Intermediate "." and ".." indicates one and several intermediate residues, respectively, not used for the sequence identity calculations. Binding site residues conserved with respect to the templates are indicated as follows: ***** conserved to GluCl; **.** conserved to the included residues from ELIC.
