# Supplementary material for: A Unified Model of the GABAA Receptor Comprising Agonist and Benzodiazepine Binding Sites
Source: PLoS One. 2013 Jan 7;8(1):e52323. doi: 10.1371/journal.pone.0052323 (PMC3538749; doi:10.1371/journal.pone.0052323)
Supplement: Figure S2 — RMSD plot and details of a 48 ns molecular dynamics simulation. (PDF) [file pone.0052323.s003.pdf]

Supporting Information – Figure S2

Stability assessment by molecular dynamics

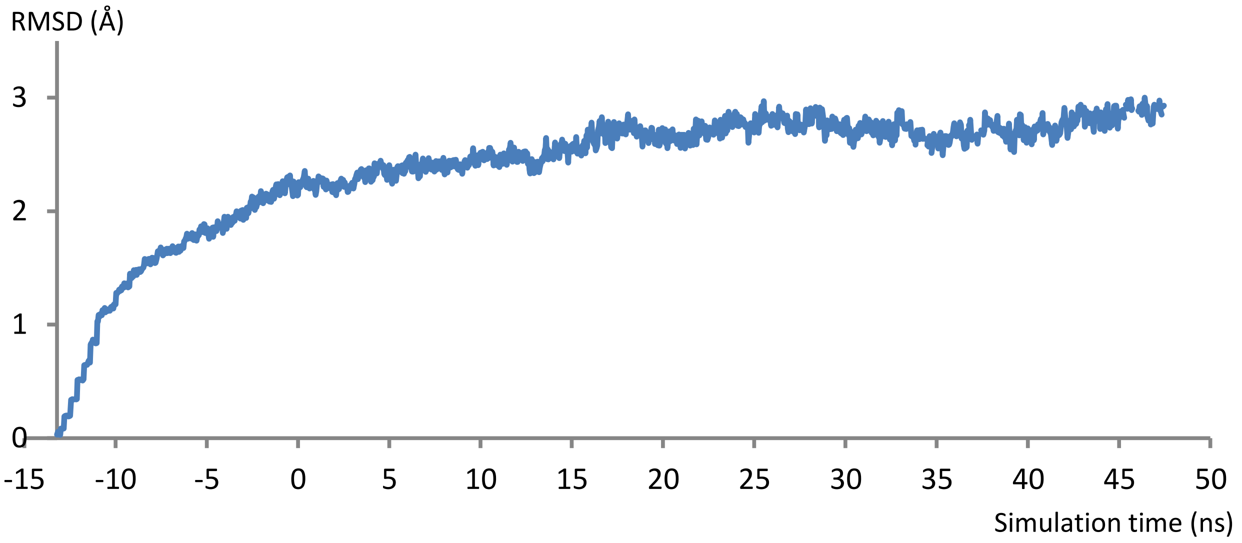

Figure S2. RMSD-plot for a 48 ns molecular dynamics simulation on the GABA$_A$R model. The root-mean-square distances from the initial model on the Cα atoms are illustrated. A multistep equilibration was performed prior to the simulation (see text for details). The production run starts at 0 ns in the figure.

Protocol

To evaluate the stability of the membrane-embedded GABA$_A$R model reported here, a 48 ns molecular dynamics (MD) simulation was run using Desmond v. 2.4 [1,2] with the following setup. The model was solvated in a two-step procedure. First, favorable positions for water molecules in the protein-ligand complex were predicted using the GRID [3,4] MINIM/FILMAP procedure where energy minimas for a water probe (OH2) were calculated and afterwards oxygen atoms inserted. A grid spacing of 0.5 Å (NPLA=2) was used and positions with energy minimas ≤ -8 kcal/mol selected. Subsequently, hydrogen atoms were added to the system, then the system was embedded in a POPC membrane and solvated in TIP3P water. Positioning of the membrane was guided by the GluCl structure (PDB ID: 3RHW) from the Orientations of Proteins in Membranes (OPM) database [5]. The Desmond System Builder was used for setting up the system with periodic boundary conditions, resulting in a box with the dimensions 126Å x 124Å x 143Å containing 210,474 atoms

and a salt concentration of 0.15 mol/L. The system was energy minimized to a convergence threshold of 1.0 kcal/mol/Å using a steepest descent method implemented in the Desmond application in Maestro v. 9.1 [6].

The system was equilibrated prior to MD production simulation using the following procedure:

1) Energy minimization of solvent and ions with a 50 kcal/mol/Å position restraint on protein and ligand heavy atoms until convergence at 50 kcal/mol/Å.
2) Energy minimization with 5 kcal/mol/Å position restraints on ligands and protein C, O and N atoms until convergence at 5 kcal/mol/Å.
3) Energy minimization without restraints to a convergence at 5 kcal/mol/Å.
4) 12 ps MD simulation at 10 K in NVT ensemble and a 50 kcal/mol/Å restraint on solute heavy atoms. Timestep of 1:1:3 fs (bonded:near:far interactions) .
5) 24 ps MD simulation as 4) but in the NPT ensemble.
6) 60 ps MD simulation as 5) but at 300 K.
7) 120 ps MD simulation, now with a 5 kcal/mol/Å restraint on solute heavy atoms.
8) 120 ps MD simulation, Cα and ligand heavy atoms restrained at 5 kcal/mol/Å and other protein heavy atoms restrained at 1.0 kcal/mol/Å.
9) 120 ps MD simulation, Cα and ligand heavy atoms restrained at 5 kcal/mol/Å.
10) 120 ps MD simulation, Cα and ligand heavy atoms restrained at 3 kcal/mol/Å.
11) 120 ps MD simulation, Cα and ligand heavy atoms restrained at 1 kcal/mol/Å.
12) 360 ps MD simulation, Cα and ligand heavy atoms restrained at 0.25 kcal/mol/Å.
13) 360 ps MD simulation, Cα restrained at 0.225 and ligand heavy atoms at 0.25 kcal/mol/Å.
14) 360 ps MD simulation, Cα restrained at 0.20 and ligand heavy atoms at 0.25 kcal/mol/Å.
15) 360 ps MD simulation, Cα restrained at 0.175 and ligand heavy atoms at 0.25 kcal/mol/Å.
16) 360 ps MD simulation, Cα restrained at 0.15 and ligand heavy atoms at 0.25 kcal/mol/Å.
17) 360 ps MD simulation, Cα restrained at 0.125 and ligand heavy atoms at 0.25 kcal/mol/Å.
18) 360 ps MD simulation, Cα restrained at 0.10 and ligand heavy atoms at 0.20 kcal/mol/Å.
19) 360 ps MD simulation, Cα restrained at 0.075 and ligand heavy atoms at 0.15 kcal/mol/Å.
20) 360 ps MD simulation, Cα restrained at 0.05 and ligand heavy atoms at 0.10 kcal/mol/Å.
21) 360 ps MD simulation, Cα restrained at 0.025 and ligand heavy atoms at 0.05 kcal/mol/Å.
22) 360 ps MD simulation without restraints.

Unless specified otherwise above, default Desmond settings were used. These are the OPLS-AA 2005 force field, the Isothermal-Isobaric ensemble (NPT), 300 K, a pressure at 1 atm, and imposing the SHAKE algorithm allowing for a time step of 2:2:6 fs (bonded:near:far interactions).

The following 48 ns production run was performed with default Desmond settings, however, in the NPγT ensemble. Frames were saved every 9.6 ps and every 4th frame was analyzed for the RMSD plot illustrated in Figure S2.

References

1. Desmond Molecular Dynamics System v. 2.4 (2010), D. E. Shaw Research, New York, NY
2. Bowers KJ, Chow E, Xu H, Dror RO, Eastwood MP, et al. Scalable Algorithms for Molecular Dynamics Simulations on Commodity Clusters; 2006 November 11-17; Tampa, Florida.
3. GRID v. 22. Molecular Discovery Ltd., 215 Marsh Road, HA5 5NE Pinner, Middlesex, UK.
4. Goodford PJ (1985) A computational procedure for determining energetically favourable binding sites on biologically important macromolecules. J Med Chem 28: 849-857.
5. Lomize MA, Lomize AL, Pogozheva ID, Mosberg HI (2006) OPM: orientations of proteins in membranes database. Bioinformatics 22: 623-625.
6. Maestro v. 9.1 (2010) Schrödinger L, New York, NY, USA.
